# Supplementary figures and images for: Progression Risk Score Estimation Based on Immunostaining Data in Oral Cancer Using Unsupervised Hierarchical Clustering Analysis: A Retrospective Study in Taiwan
Source: J Pers Med. 2021 Sep 13;11(9):908. doi: 10.3390/jpm11090908 (PMC8466609; doi:10.3390/jpm11090908)

(A) Negative control (case 1)

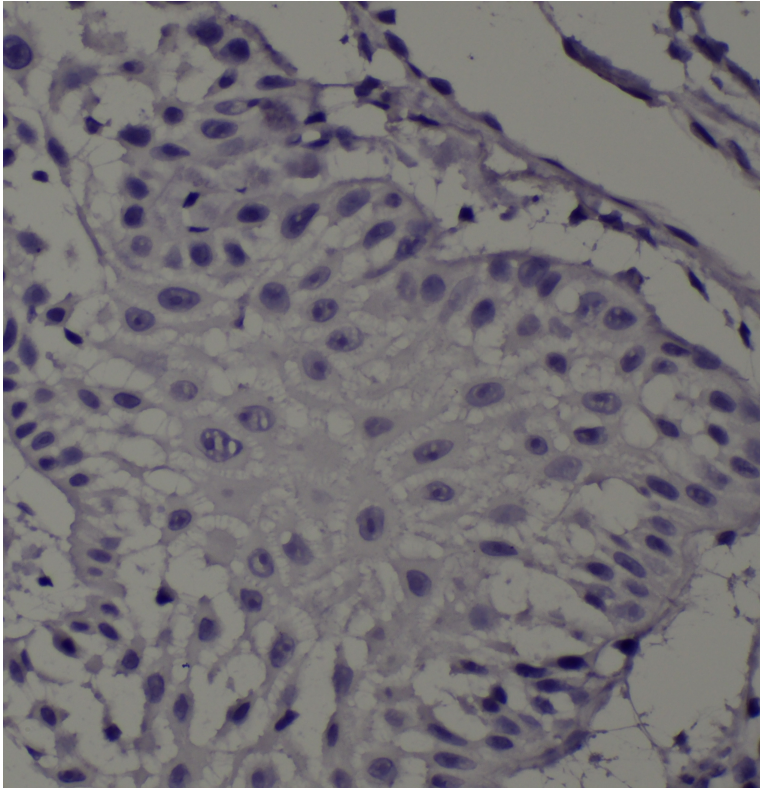

(B) Negative control (case 2)

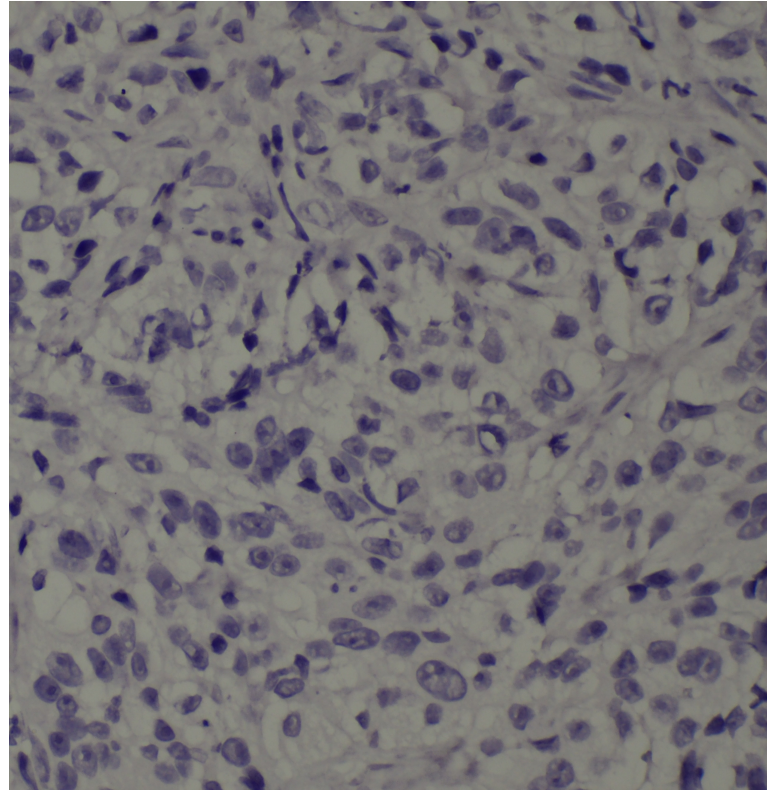

Supplement: Supplementary file 1 [file jpm-11-00908-s001.zip › jpm-1341087-supplementary.pdf]
